# Supplementary material for: Seasonal and ontogenetic variation of whiting diet in the Eastern English Channel and the Southern North Sea
Source: PLoS One. 2020 Sep 23;15(9):e0239436. doi: 10.1371/journal.pone.0239436 (PMC7511009; doi:10.1371/journal.pone.0239436)
Supplement: S1 Fig — An interpolation of A. opercularis’ δ15N values was performed to obtain baseline isotope ratios at all stations where whiting were collected (see the method in the material and method section of the manuscript). (DOCX) [file pone.0239436.s001.docx]

**S1 Fig.** Localization of whiting (orange crosses) and *Aequipecten opercularis* (green crosses) sampling stations by this, but also previous studies (Jennings & Warr 2003, P. Cresson unpubl. data). An interpolation of *A. opercularis*’ δ^15^N values was performed to obtain baseline isotope ratios at all stations where whiting were collected (see the method in the material and method section of the manuscript).
