# Supplementary material for: Seasonal and ontogenetic variation of whiting diet in the Eastern English Channel and the Southern North Sea
Source: PLoS One. 2020 Sep 23;15(9):e0239436. doi: 10.1371/journal.pone.0239436 (PMC7511009; doi:10.1371/journal.pone.0239436)
Supplement: S2 Fig — Figures A and B represented percentages of occurrence (%O) in autumn (A) and winter (B) respectively. Figures C and D represented percentages of abundance (%N) in autumn (C) and winter (D) respectively. The number of non-empty stomach content is expressed under each size class. (DOCX) [file pone.0239436.s002.docx]

**S2 Fig.** **Ontogenetic dietary changes for autumn and winter.** Figures A and B represented percentages of occurrence (%O) in autumn (A) and winter (B) respectively. Figures C and D represented percentages of abundance (%N) in autumn (C) and winter (D) respectively. The number of non-empty stomach content is expressed under each size class.
