# Supplementary material for: Seasonal and ontogenetic variation of whiting diet in the Eastern English Channel and the Southern North Sea
Source: PLoS One. 2020 Sep 23;15(9):e0239436. doi: 10.1371/journal.pone.0239436 (PMC7511009; doi:10.1371/journal.pone.0239436)
Supplement: S1 Table — HaulNum corresponds to the number of the sampling station. δ15N baseline corresponds to interpolated δ15N isotopic ratios of A. opercularis at all stations where whiting were collected. (DOCX) [file pone.0239436.s003.docx]

**S1 Table.** Geographic coordinates of the whiting sampling stations for both seasons (CGFS in autumn and IBTS in winter). HaulNum corresponds to the number of the sampling station. δ^15^N baseline corresponds to interpolated δ^15^N isotopic ratios of *A. opercularis* at all stations where whiting were collected.

| **Survey** | **HaulNum** | **Longitude** | **Latitude** | **δ^15^N baseline** |
| --- | --- | --- | --- | --- |
| CGFS | V0392 | 1.02 | 50.92 | 8.05 |
| CGFS | V0393 | 0.85 | 50.87 | 7.97 |
| CGFS | V0396 | 0.68 | 50.96 | 8.27 |
| CGFS | V0398 | 0.55 | 50.80 | 7.52 |
| CGFS | V0399 | 0.58 | 50.88 | 7.82 |
| CGFS | V0409 | 0.91 | 50.58 | 7.87 |
| CGFS | V0410 | 0.98 | 50.66 | 7.88 |
| CGFS | V0411 | 0.88 | 50.44 | 8.06 |
| CGFS | V0412 | 0.92 | 50.48 | 8.02 |
| CGFS | V0413 | 0.98 | 50.49 | 8.05 |
| CGFS | V0416 | 0.76 | 50.31 | 8.10 |
| CGFS | V0417 | 0.75 | 50.19 | 8.26 |
| CGFS | V0419 | 0.90 | 50.11 | 8.56 |
| CGFS | V0420 | 0.92 | 50.21 | 8.53 |
| CGFS | V0436 | -0.23 | 49.56 | 8.49 |
| CGFS | V0459 | -0.27 | 50.58 | 7.48 |
| CGFS | V0471 | -0.69 | 49.42 | 8.56 |
| CGFS | V0472 | -0.80 | 49.51 | 7.89 |
| CGFS | V0477 | -0.24 | 49.46 | 8.80 |
| CGFS | V0479 | -0.57 | 49.45 | 8.57 |
| IBTS | W0001 | 0.86 | 50.33 | 8.28 |
| IBTS | W0002 | 0.71 | 50.34 | 7.90 |
| IBTS | W0003 | 0.48 | 50.38 | 7.65 |
| IBTS | W0011 | 0.12 | 49.84 | 7.57 |
| IBTS | W0012 | 0.25 | 50.30 | 7.22 |
| IBTS | W0013 | 0.22 | 50.54 | 7.53 |
| IBTS | W0019 | 0.47 | 50.56 | 7.52 |
| IBTS | W0021 | 0.95 | 50.78 | 7.86 |
| IBTS | W0029 | 1.13 | 51.02 | 8.22 |
| IBTS | W0030 | 1.37 | 51.12 | 8.31 |
| IBTS | W0031 | 1.42 | 51.39 | 8.26 |
| IBTS | W0140 | 1.31 | 51.33 | 8.29 |
| IBTS | W0141 | 1.08 | 51.27 | 8.37 |
| IBTS | W0142 | 0.78 | 51.05 | 8.33 |
