# Supplementary material for: Seasonal and ontogenetic variation of whiting diet in the Eastern English Channel and the Southern North Sea
Source: PLoS One. 2020 Sep 23;15(9):e0239436. doi: 10.1371/journal.pone.0239436 (PMC7511009; doi:10.1371/journal.pone.0239436)
Supplement: Attachment — Submitted filename: Response_to_Reviewers.docx [file pone.0239436.s004.docx]

**Journal Requirements:**

***1.*** *Thank you for including your ethics statement:  “Animals were collected during IFREMER annual surveys. Protocol was validated by chief scientist of the survey”*

*To comply with PLOS ONE submissions requirements, please provide the following information in the Methods section of the manuscript and in the “Ethics Statement” field of the submission form (via “Edit Submission”):*

*a.    Please amend your current ethics statement to include the full name of the ethics committee that approved your specific study.*

*b.    Please amend your current ethics statement to confirm that your named ethics committee specifically approved this study.*

The following sentence was included in the material and methods section of the manuscript:

L. 99-102 (on the manuscript without track changes): « Protocols of all surveys are currently being evaluated by the French research institute for exploitation of the sea (Ifremer) and are validated by the ICES IBTS International Group [41]. In addition, survey's PIs received training about animal well-being and ethics. »

*For additional information about PLOS ONE submissions requirements for ethics oversight of animal work, please refer to http://journals.plos.org/plosone/s/submission-guidelines#loc-animal-research*

***2.*** *In your Methods section, please provide additional location information of the survey sites, including geographic coordinates for the data set if available.*

Locations of the sampling stations are represented In Figure 1, with actual coordinates displayed in the supplementary appendix 1B and in the dataset available at the following address: <https://doi.org/10.17882/72233>

L. 95-96: “Sampling locations are presented in the map (Fig. 1), and actual coordinates are presented in the supporting materials (S1 Fig. and Table)

***3.*** *To comply with PLOS ONE submissions requirements, please provide methods of sacrifice in the Methods section of your manuscript.*

Organisms were sacrificed following the standard international IBTS protocol, validated by the dedicated ICES international working group. This clarification was included in the material and method section of the manuscript.

L. 110-111: “Organisms were sacrificed following the standard international IBTS protocol [41].”

**Additional Editor Comments:**
 *I have received the comments from two expert reviewers. Although reviewer #2 is relatively positive, both reviewers raised several critical concerns.*

***1.*** *For example, reviewer #1 commented that SI data of predators cannot be interpreted appropriately without those of prey or food-web baseline. As a result, the discussion is speculative or not well integrated. This is a common concern that is apparent prior to research. You may address this problem by focusing on the size dependence of feeding habits under the assumption that all size classes have the same baseline (if this is truely reasonable) or even by removing SI data from the manuscript*.

The bivalve *Aequipecten opercularis* (queen scallops) was chosen as baseline, following most classical approach (e.g., Jennings and van der Molen 2015, reference 20 in the plain text). Since *A. opercularis* and whiting were not always collected in the same stations, a geostatistical interpolation (namely kriging) of the baseline stable isotope ratios were performed in order to consider its spatial variation. Interpolation is based on isotopic ratios, (1) measured on samples collected in the present work simultaneously with fish, (2) measured during previous projects and unpublished yet and, (3) retrieved from literature (Jennings and Warr 2003). The following section detailing the interpolation method was added to the material and methods section of the manuscript.

L. 168-177: “A station-specific baseline value was used to take into account the spatial variability of isotopic ratios (S1 Fig and Table). As *A. opercularis* samples could not always be found in the stations where fish were collected, missing δ^15^N values were calculated with a geostatistical interpolation of baseline δ^15^N values developed by Kopp et al. [30], and using *A. opercularis* isotopic ratios obtained in the present and previous published [44] and unpublished sudies (P. Cresson unpubl. data). The dependency of covariance on distance between two sampling sites was modelled using a non-linear regression based on weighted least squares. The weight is the number of pairs of points per distance intervals. Spatial covariance in δ^15^N was best described by a Gaussian model of distance with adjR2= 0.99.”

Furthermore, the spatial variation of *A. opercularis* δ^15^N ratios, whether measured or interpolated, is now presented in a map added in the supplementary appendix, making it possible to estimate a baseline value at each station where whiting were collected. The predicted values are presented in the table of the supplementary table 1 (S1 Table). The inclusion of the baseline allowed us to calculate the trophic level of each individual, *i.e.,* providing an absolute value that takes the spatial variability into account.

However, since we do not have input data for some coastal sampling stations or some stations with local specificities (*e.g.* fish farm neighborhood), the accuracy of the values interpolated at these stations may be questioned, leading to a potential overestimation of some trophic level values, particularly for smallest individuals collected in winter. A cautious point was added in the discussion section of the manuscript.

L. 279 – 284:” Previous studies based on stable isotopes confirmed the important benthic-pelagic coupling in this area, but were not able to identify mechanisms at play, *i.e.,* if coupling results from the consumption of benthic invertebrates by pelagic fish or from the integration of benthic production in the pelagic pathway [22,30]”.

L. 410 – 419: “Unfortunately, no information on isotopic ratios of coastal queen scallops could be collected as the survey trawl could not be operated in shallow waters. Thus, as kriging is directly dependent upon input data, local influences on queen scallops isotopic ratios could not be efficiently captured, and may have induced an overestimation of small whiting δ^15^N values and trophic levels. Interestingly, estimation of trophic level was associated with highest uncertainties at coastal locations in the model of Jennings and van der Molen [20], as a result of the effect of salinity on baseline δ^15^N ratios. The high concentration of juveniles in coastal areas, and their limited movement abilities may explain why this influence of terrigenous or fish farm derived nitrogen would primarily affect the smallest size class.”

***2.*** *Also, I agree with Reviewer #1 that you may consider quantitative data on predator body size rather than categorizing them into different size classes. I suggest that re-analyses of the data would be necessary.*

GLM analyses were performed with both continuous fish sizes and discrete-size categories. Final analyses considered discrete size classes for several reasons:

(1) For stomach contents analyses, size-class analyses allow to account for the highly non-linear character of dietary mechanisms. Each individual within a size class is considered as a replicate of the class. Having the diet of some individuals within a size-class can be viewed as a way to capture the average diet of a class, and to limit the importance of extreme feeding mechanisms. This approach is typically used when ontogenetic feeding changes are studied using stomach contents (see by example Graham et al. 2007, Demain et al. 2012, Ross et al. 2016, references 37, 25 and 24 in the plain text).

(2) Using discrete size classes in isotopic analyses GLMs was also justified a posteriori by the non-monotonic character of the isotopic ratios versus size relationships.

The Table below presents for information the GLM results obtained using continuous fish sizes (not shown in the MS).

| **Response variable** | **df** | **Residual deviance** | **Explanatory variables** | **df** | **Deviance** | **Residual deviance** | **df** | **p-value (Chi-square)** |
| --- | --- | --- | --- | --- | --- | --- | --- | --- |
|  |  |  |  |  |  |  |  |  |
| **Occurrence – fish** | 245 | 308.47 | **Size class** | **1** | **17.92** | **323.04** | **244** | **<0.001** |
|  |  |  | **Season** | **1** | **5.44** | **611.30** | **243** | **0.02** |
|  |  |  | **Size class × season** | **1** | **9.13** | **587.80** | **242** | **0.003** |
| **Occurrence - Benthic crustaceans** | 245 | 304.06 | Size class | **1** | **6.66** | **321.51** | **244** | **0.01** |
|  |  |  | Season | 1 | 3.19 | 318.32 | 243 | 0.07 |
|  |  |  | **Size class × season** | **1** | **14.27** | **304.06** | **242** | **<0.001** |
| **Occurrence - Pelagic crustaceans** | 245 | 225.50 | **Size class** | **1** | **68.33** | **247.54** | **244** | **<0.001** |
|  |  |  | **Season** | **1** | **20.32** | **227.22** | **243** | **<0.001** |
|  |  |  | Size class × season | 1 | 1.72 | 225.50 | 242 | 0.19 |
| **Occurrence -  Unidentified crustaceans** | 245 | 303.15 | Size class | **1** | **4.77** | **306.99** | **244** | **0.03** |
|  |  |  | Season | 1 | 2.30 | 304.69 | 243 | 0.13 |
|  |  |  | Size class × season | 1 | 1.55 | 303.15 | 242 | 0.21 |
| **Occurrence - Mollusks** | 245 | 196.92 | **Size class** | **1** | **9.85** | **198.47** | **244** | **0.002** |
|  |  |  | Season | 1 | 1.36 | 197.11 | 243 | 0.24 |
|  |  |  | Size class × season | 1 | 0.20 | 196.92 | 242 | 0.66 |
| **Abundance - fish** | 245 | 291.90 | **Size class** | **1** | **25.68** | **313.18** | **244** | **<0.001** |
|  |  |  | **Season** | **1** | **12.78** | **300.40** | **243** | **<0.001** |
|  |  |  | **Size class × season** | **1** | **8.50** | **291.90** | **242** | **0.004** |
| **Abundance - Benthic crustaceans** | 245 | 2069.0 | **Size class** | **1** | **390.59** | **2146.5** | **244** | **<0.001** |
|  |  |  | **Season** | **1** | **77.17** | **2069.4** | **243** | **<0.001** |
|  |  |  | Size class × season | 1 | 0.37 | 2069.0 | 242 | 0.54 |
| **Abundance -  Pelagic crustaceans** | 245 | 3411.9 | **Size class** | **1** | **3344.3** | **7874.0** | **244** | **<0.001** |
|  |  |  | **Season** | **1** | **4394.2** | **3479.7** | **243** | **<0.001** |
|  |  |  | **Size class × season** | **1** | **67.8** | **3411.9** | **242** | **<0.001** |
| **Abundance - Unidentified crustaceans** | 245 | 587.80 | **Size class** | **1** | **68.47** | **683.57** | **244** | **<0.001** |
|  |  |  | **Season** | **1** | **72.28** | **611.30** | **243** | **<0.001** |
|  |  |  | **Size class × season** | **1** | **23.50** | **587.80** | **242** | **<0.001** |
| **Abundance - Mollusks** | 245 | 254.58 | **Size class** | **1** | **37.96** | **256.02** | **244** | **<0.001** |
|  |  |  | Season | 1 | 0.05 | 255.97 | 243 | 0.82 |
|  |  |  | Size class × season | 1 | 1.38 | 254.58 | 242 | 0.24 |
| δ**^13^C** | 211 | 70.77 | **Size class** | **1** | **1.63** | **76.77** | **210** | **0.03** |
|  |  |  | **Season** | **1** | **3.52** | **73.25** | **209** | **0.002** |
|  |  |  | **Size class × season** | **1** | **2.48** | **70.77** | **208** | **0.007** |
| δ**^15^N** | 211 | 160.89 | Size class | 1 | 1.45 | 273.88 | 210 | 0.17 |
|  |  |  | **Season** | **1** | **49.37** | **224.52** | **209** | **<0.001** |
|  |  |  | **Size class × season** | **1** | **63.63** | **160.89** | **208** | **<0.001** |
| **Trophic level** | 211 | 13.92 | Size class | 1 | 0.008 | 22.41 | 210 | 0.75 |
|  |  |  | **Season** | **1** | **3.99** | **18.42** | **209** | **<0.001** |
|  |  |  | **Size class × season** | **1** | **3.09** | **15.34** | **208** | **<0.001** |

***3.*** *Reviewer #2 suggested that all the descriptions about optimal foraging theory should be removed, which I totally agree with.*

We have removed all references to the optimal foraging theory in the manuscript. We replaced these assumptions by Werner and Gilliam's hypothesis (1984) related to the trade-off between foraging and predation risk. In the introduction section of the manuscript, we have rewritten the part related to ontogenetic dietary changes. In the rest of the manuscript, all parts concerning the optimal foraging theory have been deleted.

L. 33 – 40: “Organism’s energetic requirements differ according to their size. Ontogenetic dietary shifts, the changes in resource use over the lifespan of a consumer, are widespread for many taxa [10,11]. Consumers make trade-offs between foraging and mortality risk [10]. These trade-off changes according to ontogeny depending on the balance between the risk of mortality due to predation and the benefits received from the resource. Several biotic (predation risk, competition, prey availability) or abiotic (habitat use) factors may be responsible of these variations [12]. In addition, species intrinsic factors, such as the gape size or swimming abilities promote these ontogenetic changes.”

*In addition, I found other concerns based on my own reading. Please consider the followings as well.*

***4.*** *L147-152: These results are natural because the sample sizes are almost constant across size classes (i.e., the sample sizes do not reflect size distribution).*

We agree with the reviewer, and this was actually a purpose of this sampling. The sampling was not designed to represent the natural variability of the size distribution, but to capture the size-driven variability in diet. So, as stomach content analysis requires more samples than stable isotope analysis to statistically examine differences among size class (see by example Hyslop 1980, Cresson et al. 2014, references 43 and 38 in the manuscript), the sampling was refined. The representativeness of the subsample was confirmed by the similarity in average size between the whole dataset and the subsample used for stable isotope analysis. A sentence detailing this point was added:

L. 203-204: “Subsample used for stable isotopes analyses can thus be considered representative of the larger sample used for stomach content analyses.”

***5.*** *L158-167: The results of taxon-specific %N are not presented anywhere (the same is true for some parts of Discussion).*

A figure representing %N was added in the supplementary appendix of the manuscript (S2 Fig.). For each individual the taxon-specific results are presented in the dataset presented at the following address: <https://doi.org/10.17882/72233>

L. 218 – 219: “The taxon-specific results can be found in the dataset published at the following address: <https://doi.org/10.17882/72233>.”

***6.*** *L210-: Many parts of Discussion have argued that the present findings are consistent with previous ones. If so, I suggest that such information should be provided in Introduction. Then, please state the novelty of this work more clearly.; i.e., what has been done and remains to be done in this system or other similar systems (e.g., the same species/other species in this/other areas) in Introduction.*

The trophic ecology of whiting has been studied in several ecosystems. However, all biotic and abiotic conditions between ecosystems can lead to differences in feeding habits. In the EEC-SNS, the trophic ecology of whiting was mainly studied by stomach contents analyses. A Previous study using stable isotope analyses have not investigated the extent to which it was affected by ontogenetic and seasonal changes (see by example Kopp et al. 2015, reference 30 in plain text). In this study, we propose to study whiting trophic ecology using a combination of isotopic and stomach content analyses to provide information at several time scales. Our sampling allows us to study ontogenetic variations and to consider seasonal differences of whiting diets. We have added a paragraph in the introduction section of the manuscript on this subject:

L. 56 – 74: “The diet of this high trophic level species is well investigated in many ecosystems because it predates on commercially exploited fish and invertebrates, and also since it can compete with other economically valuable gadoid species [24]. Most studies highlight the main consumption of fish and invertebrates (e.g. crustaceans, mollusks or annelids). Some of them report an ontogenetic change in whiting diet, with small individuals mostly consuming crustaceans, and larger whiting mostly consuming fish. This feeding pattern has been previously observed in other ecosystems, *i.e.*, the North Sea [24–27], Baltic Sea [24], Irish Sea [28] and Celtic Sea [27,29]. However, ecosystems are subject to different environmental and ecological conditions. All differences in prey availability, predator abundance, fishing pressure and abiotic features make differences between the ecological role and diet of *M. merlangus* in an ecosystem and its conspecific in other ecosystems. To our knowledge, studies that have investigated EEC whiting diets have not focused on ontogenetic and seasonal diet changes [30,31]. The increased trophic importance of fish with ontogeny is consistent with outputs of an independent modeling study based on an EEC application of the OSMOSE ecosystemic model [15]. Except this modeling study, the trophic ecology of whiting in EEC-SNS was mainly studied by stomach content analyses, providing a short-term view of its diet [24,25,31]. The use of stable isotope analyses in addition to analyses of stomach contents helps to resolve some common biases related to the analysis of stomach contents.”

***7.*** *L292-: It is not surprising that SI data are not consistent with SC data, as you have supposed in M&M (L109-113). Although you have discussed several hypotheses, another plausible explanation would be that SC data could be more reliable if prey biomass is considered. You may discuss this possibility.*

We recognize that consideration of prey biomass would enable a better understanding of trophic ecology, and would allow the integration of additional indices such as the index of relative importance taking into account both abundance, occurrence, and prey biomass. However, although fish were immediately frozen after capture to limit digestion, many preys were too degraded to estimate their biomass accurately. This may question the relevance of using biomass as a reliable descriptor of fish diet (Baker et al. 2014). Biomass of remaining hard pieces (crustacean claws, fish otoliths etc.) cannot be easily converted into the actual biomass of the original prey consumed without accurate mass-mass or length-mass relationships. Including biomass as a descriptor of fish diet would also increase the well-known bias due do different prey digestibility, since the ratio between actual mass (i.e. the requested information) and measured mass of prey at different stages of degradation would not be similar,

For this reason, we only considered in our analyses prey occurrence and abundance.

*Overall, I suggest that almost all parts of the manuscript should be rewritten. I recognize that revision efforts will be too much, but I basically encourage publications of such descriptive information of behavior and ecology. I hope you to consider all the comments carefully for resubmission.*

**Reviewer #1’s comments**

*This manuscript researches ontogenetic and seasonal diet change of whiting in the Eastern English Channel and Southern North Sea. Diet components of the fish were investigated from stomach contents and stable isotope analysis from 248 and 212 samples, respectively. The relationship between diet (presence or absence of each diet item for SCA and 13C and 15N values for SIA) and explanatory variables (body size classes and season) was assessed by Generalized Linear Models (though it was unclear why they divided their data into 6 age classes without using body size for individual fish). Stomach contents analysis revealed that the frequency of fish increased with fish size, whereas that of pelagic crustaceans decreased vice versa. Although authors claimed that their SIA data somewhat agreed with that of their SCA, they appeared at a loss over how to interpret their results in relation to the SIA, especially for larger fish.

The methods and discussion related to SCA seem elaborate and well thought out.*

***1.*** *However, the SIA of this study critically lacks important data for proper interpretation of them. Please be aware that SI data for bulk tissue of marine organisms cannot be properly interpreted without understanding of isotope ratios of potential diet and baseline. Trophic position of target fish is nothing more than one of a potential factors to determine their isotope ratios, but baseline shift often results in larger isotopic variation than dietary shift (see Reddin et al. 2017). Specifically, the habitat of studied species is likely to involve high variation in isotopic baseline, due to a large difference in the water depth within the region. Therefore, SI data presented in this study are intrinsically uninterpretable in current form and do not support any results of SCA.*

The point you raise has also been mentioned by the editor. You will find an answer to this point in the first comment from the editor (see above).

***2.*** *Another issue in SIA of this study is that the authors assumed the same turnover time of muscle among body size classes. In the case of soft tissues like muscle, isotope turnover includes two different factors; metabolic tissue replacement and incremental growth of the tissue. Generally, young fish have high growth rate and active incremental growth of muscle results in their rapid turnover time, whereas old fish are only influenced by metabolic tissue replacement and thus their turnover tend to be slow (see Vander Zanden et al. 2015). In addition, turnover time often differ between 13C and 15N; 13C generally have higher turnover rate than 15N. Given such an inconsistent turnover in muscle among growth stages of whiting, I wander the seasonal comparison of this study is really meaningful.
On the basis of these shortcomings in SIA of this study, I cannot recommend to publish this paper in its current form. I urge you to rewrite the manuscript by taking into account the isotope baseline and variation in turnover time of fish with different growth stage, or completely remove the description related to SIA from the paper.*

Turnover rate is often expressed as isotopic half-life, defined as the time required reaching 50% equilibration with diet. Vander Zanden et al (2015) have developed equations to estimate the turnover rate, which varies according to the type of organism (endotherm *vs*. ectotherm) and the type of tissue. For fish muscle, the equation is as follows:

ln(half life) = 0.21 . ln( body mass) + 3.23

In our study, whiting belonging to the smallest size class have an average mass of 15g. Those in the largest size class have an average mass of 508g. By applying the above equation, we can then estimate a turnover of about 45 days for the smallest individuals and 93 days for the largest, i.e., about three months. Considering a turnover rate of several months for adults is classical for isotope analyses (see for example Hesslein et al., 1993, McIntyre & Flecker, 2006, Nielsen et al., 2018, references 86, 87 and 82 in the plain text).

We then admit that juveniles’ isotope ratios may reflect feeding during the collection season (autumn or winter). For adults, since the turnover time is estimated to be about three months, this may correspond to feeding prior to the collection season.

In the discussion, we added a section on the potential differences of turnover between juveniles and adults. We thank the reviewer for making us think about this possibility.

L. 418 – 446 : “Stomach content analysis provides short-term information about the integrated diet, from hours to days [43,82]. Stable isotopes analysis provides information on assimilated diet at a longer time scale than stomach contents, but varies according to the lifecycle of species. A meta-analysis carried out on several taxa revealed that, in general, turnover rate differs between juveniles and adults. Stable isotopes ratios change due to growth and metabolic replacement associated with a change in diet were observed between juveniles and adults [83]. These authors have developed an equation to estimate the turnover rate according to the body mass (proxy of the size) and to the type of species (endotherm vs. ectotherm) and tissue. Tissues characterized by a quick turnover provide the most recent dietary information. Using this equation, the turnover rate was estimate to ~45 days for juveniles (15 g average mass) which is consistent with the turnover rate estimated for cod, another gadoid species [84]. We then admit that juveniles ‘isotope ratios may reflect feeding during the collection season (autumn or winter). For adults (weighing 508 g on average), the turnover rate was of about three months, consistently with the expected turnover of adult fish [82,85,86]. Measured isotopic ratios may partly result from dietary elements integrated in the tissues before the sampling season, and away from EEC, due to movement abilities of adult whiting [87]. However, isotopic integration of diet has to be viewed from a dynamic point of view. Addressing the seasonal (*i.e.* in autumn and winter) and spatial (*i.e.* over the potential area covered by whiting over a three months period) variability of prey isotopic ratios would also be needed. Finally, since the integration of isotopes in tissues is dynamic, additional studies are necessary to know precisely the rate at which isotopes are incorporated into whiting’s muscle and the discrimination factor between tissues and diet [88]. It should also be noted that the method used to estimate turnover rates does not distinguish between isotopes (δ^13^C, δ^15^N). However, several studies have shown that for fish, the turnover rate of δ^13^C is faster than that of δ^15^N due to different catabolic processes operating on the various biochemical constituents of tissues (amino acids, proteins, lipids [89–91]). Consequently, both δ^13^C and δ^15^N for the same species inform about diet at different time scales, highlighting the importance of considering the turnover rate to know when that diet has been assimilated for a better understanding of species diet.”

*Detailed comments line by line:*

***3****. Table 1: Why predicted mean isotope ratios completely agreed with measured values?*

This pattern results from the fact that Isotopic ratios were analyzed using a standard linear model with a Gaussian error structure. This would not necessarily be true with Generalized Linear Models and using alternative error types. We illustrate below how predictions were calculated.

Considering that the prediction (Y) = A + B*_i_* + C*_j_* + D*_i,j_* with

A= intercept, B*_i_* = estimate for the size class *i* + C*_j_* = the estimate of the season *j* and D*_i,j_* the interaction between size class *i* and season *j*.

Below is an example for the predicted δ^15^N values

Prediction for the 20-25cm size class in winter:

Y = 14.4194 + 1.6028 + 0.4151 + 0.2250 = 16.66

We removed predicted mean isotope ratios in the manuscript

**4.** Line 120: This is incorrect equation. Add “(‰)” after “δX”.

Done

***5.*** *Line 129: Just using fish size as explanatory variable (not size classes) is better way for this analysis. Using size classes instead of actual size results in degraded levels of measurement of your data.*

This point has also been mentioned by the editor. See the answer to this point in the second comment from the editor (see above).

***6.*** *Lines 235-236: Although stable carbon isotope ratios are conventionally used as pelagic food web tracers, no studies have tested general applicability of such use of 13C. In the case of the North Sea, spatial distribution of 13C values are clearly inconsistent with that of pelagic water (see Trueman et al. 2017).*

We agree with the reviewer: due to the relative nature of stable isotopes, an isotopic ratio *per* *se* cannot be considered as tracer of pelagic or benthic food web.

Nevertheless, several papers, in the EEC and other ecosystems, have demonstrated that the comparison of isotopic ratios can be used to track the relative importance of benthic or pelagic pathways, notably as carbon isotopic ratios are largely different between benthic and pelagic pathways (~2 to 3 ‰ in the EEC; Kopp et al. 2015; Cresson et al. in press) and are largely higher than the spatial variability. Increasing importance of benthic sources results in higher (less negative) isotopic ratios. In the above-mentioned sentence, we interpreted the increase in isotopic ratios with size from a relative point of view, i.e., assuming an increased contribution of benthic pathway in the diet of larger whiting individuals. We understand that this assumption was not clear enough. The sentence was thus modified in the MS as follows:

L. 290 - 292: “Since pelagic organisms are generally δ^13^C-depleted compared to benthic ones [30,35] the increase of δ^13^C values is thus consistent with a diet switch from pelagic invertebrates to benthic fish as whiting grows.”

***7.*** *Lines 258-259: I bet the result reflected migration ability of the fish. Overall, the study site is shallow and will have high 15N value, because denitrification in sediments increase the 15N value of pore water, and phytoplankton intake the 15N-enriched nitric acid. Therefore, smaller fish with low migration ability had higher 15N values. On the other hand, some large fish with high migration ability came from outside the shallow sea area will have lower 15N values. An isoscape of 15N (Trueman et al. 2017) of this area support this explanation.*

We thank the reviewer for this hypothesis. We have added it to the discussion

L. 312– 326: “The study site is shallow and presumably has high δ^15^N value, because denitrification in sediments increase the δ^15^N value of pore water, and consequently of the organic matter at the base of food webs [64,65]. Therefore, smaller fish with low migration ability had higher δ^15^N values. On the other hand, since isotopic ratios of the baseline vary spatially [44,48], we can hypothesize that the decrease in δ^15^N for larger individuals does not reflect a different diet, but an artifact related to a spatially different baseline. As mentioned by Reddin et al. [49], from an isotopic point of view, baseline can sometimes be more variable than the diet. Here, the difference between the minimal and maximal δ^15^N values kriged for queen scallops was of ~1.5 ‰ (S1 Table). However variations may be more important if considering neighboring areas, *i.e.* the Western English Channel or northern areas in the North Sea [44,48]. Assuming high migration abilities for larger whiting, their isotopic ratios may reflect a diet in areas where baseline δ^15^N is markedly lower. Investigating isotopic ratios of whiting over a large spatial continuum, with samples from the whole English Channel and the North Sea could thus be useful to better understand the influence of spatial migration on fish isotopic ratios.”

**Reviewer #2’s comments**

*In this paper the authors study the effect of ontogeny and season on the diet of whiting fish. They use two complementary methods that provide quantification over short-term feeding – stomach content analyses, and over longer term, stable isotope analyses, which provide support for ontogenetic diet shift from crustaceans to fish diet. Overall, I think this study provides interesting information on the feeding ecology of this species.
I have a couple general comments:*

***1.*** *I would suggest refraining to make any statement that this study’s results are consistent optimal foraging theory. The authors use the idea that, according to OFT, the diet selected should “maximize the energy intake per unit handling time” (l.248). This may be true, but this aspect of whiting’s ecology is not investigated at all in this study. The authors only make the assumption that the observed ontogenetic diet shift can be explained from this statement, but their data do not provide any support to this claim.*

This point has also been mentioned by the editor. See the answer to this point in the third comment from the editor (see above).

***2.*** *In the current form, the results of the stable isotope analysis are a little hard to interpret for someone with not much background on stable isotope analysis. It would be nice if the authors add a few sentences to explain briefly what general information 13C and 15N content provide about a species’ ecology. Right now the authors mostly present the result of isotope analysis as being “consistent” with stomach content analysis (except for 15N pattern in winter). Is that all this analysis does, confirming the results of stomach content analysis? Or does it also indicate something more?*

Although explanations of the δ^13^C and δ^15^N values were present in the material and methods section of the previous version, as well as the complementary vision between isotopes and stomach content analyses, these explanations may not have been sufficiently clear or emphasized. We then added a new paragraph on this subject in the introduction section of the revised version of the manuscript .Indeed, isotope analyses do not only confirm or not the results of stomach contents analyses, but also provide additional information at different time scales. We thank the reviewer for pointing this.

L. 75 - 85: “When consuming a prey, a predator integrates the carbon and nitrogen isotopic ratios of its prey into its own tissues, with a difference called isotopic discrimination [32]. The nitrogen isotopic ratio (δ^15^N), theoretically enriched by ~3.4 ‰ per trophic level, is classically used as an estimator of the trophic position [33]. Carbon isotopic ratio (δ^13^C) is less enriched (~1 ‰ per trophic level), allowing its use as tracer of the origin of food sources [34]. In the marine environment, δ^13^C is used to distinguish between benthic and pelagic sources because benthic producers are typically ^13^C-enriched relative to pelagic producers [35]. Unlike stomach content analyses, stable isotope ratios of an organism’s tissues provide information on the time-integrated assimilated diet. Combining stable isotopes and stomach content analyzes is thus recognized as a powerful approach to gain accurate and complementary vision of fish trophic patterns at different time scales [36–38].”

In addition, as raised by reviewer 1 (second comment), differences of turnover rates between juveniles and adults can provide information on diet at different time scales. See above, the second response to reviewer 1 for more explanation about this point.

*Other comments:*

***3.*** *L171-172: “As whiting grows, it consumes more fish, both in occurrence and abundance.” Looking at figure 4, it indeed seems true during winter, but not during fall, as class 15-20 cm, the second smallest class, has the highest Oi, and Oi remains more or less constant in bigger size class. For abundance, it is individuals between 15-25 cm that feed the most on fish.*

A clearer distinction was achieved between autumn and winter. Indeed, the increase in fish consumption, both in abundance and occurrence, mainly concerns the winter season. In autumn, it was mainly an increase of the mollusks consumption.

***4.*** *L287: typo: “forage”. Do you mean forager? Foraging?*

The term « forage » was removed

***5.*** *Table 1: I don’t really see the point of the columns on predicted values of δC13 and δC15. The predicted mean values are exactly the same than the observed ones, only the SD changes. Besides, I’m wondering if the SD values reported there may not be in fact estimates of the standard error, as it is the SE usually reported in GLM model analyses… and SD should not change anyway…*

The same remark was made by the reviewer 1. An explanation of the predicted values can be found above (third comment of the reviewer 1). Since the predicted values are provided from glm, the term « SD » has been replaced by "SE", more appropriate for model outputs.

***6.*** *Fig 3.A: The maximum size class is 35+, so I am not sure why the graph continues beyond that point… In fig 3B, this is where it stops…*

This was just a graphical concern on the x-axis. The maximum size class is 35+, and that is where the graph stops, as in Figure 3B. This problem has been solved.

***7.*** *Fig 3. And 4A: It took me some time to figure out that the difference in the data plotted is that Fig 3 (%O) and fig 4A (Oi), make it more explicit in figure’s legends. They are essentially the same data presented differently, so I wonder if Fig. 4A is truly necessary. Also the Y-axes should be labelled*

In order to avoid redundancies between figure 3 and figure 4, we have moved figure 3 to supplementary appendix (S2 Fig).

Figure 4a shows the probability of occurrence for each size class. Results provided from GLM models. Y-axes were respectively relabeled « Probability of occurrence » and « Number of prey items (N_i_) respectively »

***8.*** *Figure 4: Is the “error bar” SD or SE*

For Figure 4, since data presented are the result of GLM models, error bars corresponds to SE. This information was added in the legend of the figure

***9.*** *Figure 4: The bar from the 10-15 cm class in winter should be completely removed, as n=0.*

Done

***10.*** *Figure 4 – plot of pelagic crustaceans abundance: the value of the 15-20cm in winter is very high making it impossible to see what’s going on for all the other measurements. I think the authors should make one of those graphs with a break on the y-axis, from e.g. 10 to 60 cm.*

Done

***11.*** *Figure 4. typo on the lowest-left graph: “Undicentified crustaceans”*

The typo was corrected on the graph

***12.*** *Fig. 5 legend should make reference to the color that corresponds to the season.*

Done
