# Supplementary material for: Seasonal and ontogenetic variation of whiting diet in the Eastern English Channel and the Southern North Sea
Source: PLoS One. 2020 Sep 23;15(9):e0239436. doi: 10.1371/journal.pone.0239436 (PMC7511009; doi:10.1371/journal.pone.0239436)
Supplement: Attachment — Submitted filename: Response_to_reviewers.docx [file pone.0239436.s005.docx]

PONE-D-20-06925R1
Seasonal and ontogenetic variation of whiting diet in the Eastern English Channel and the Southern North Sea
PLOS ONE

Dear Dr. Timmerman,

Thank you for submitting your manuscript to PLOS ONE. After careful consideration, we feel that it has merit but does not fully meet PLOS ONE’s publication criteria as it currently stands. Therefore, we invite you to submit a revised version of the manuscript that addresses the points raised during the review process.

Please submit your revised manuscript by Sep 06 2020 11:59PM. If you will need more time than this to complete your revisions, please reply to this message or contact the journal office at plosone@plos.org. When you're ready to submit your revision, log on to https://www.editorialmanager.com/pone/ and select the 'Submissions Needing Revision' folder to locate your manuscript file.

Please include the following items when submitting your revised manuscript:

- A rebuttal letter that responds to each point raised by the academic editor and reviewer(s). You should upload this letter as a separate file labeled 'Response to Reviewers'.
- A marked-up copy of your manuscript that highlights changes made to the original version. You should upload this as a separate file labeled 'Revised Manuscript with Track Changes'.
- An unmarked version of your revised paper without tracked changes. You should upload this as a separate file labeled 'Manuscript'.

If you would like to make changes to your financial disclosure, please include your updated statement in your cover letter. Guidelines for resubmitting your figure files are available below the reviewer comments at the end of this letter.

If applicable, we recommend that you deposit your laboratory protocols in protocols.io to enhance the reproducibility of your results. Protocols.io assigns your protocol its own identifier (DOI) so that it can be cited independently in the future. For instructions see: [http://journals.plos.org/plosone/s/submission-guidelines#loc-laboratory-protocols](http://journals.plos.org/plosone/s/submission-guidelines#loc-laboratory-protocols)

We look forward to receiving your revised manuscript.

Kind regards,

Takefumi Nakazawa
Academic Editor
PLOS ONE

**Additional Editor Comments (if provided):**

I have received the comments from the same reviewers. Both of them appreciated your revision efforts, but they addressed several issues. I hope you to consider all the comments carefully for resubmission.

In addition, I found other concerns based on my own reading. Please consider the followings as well.

**1.** I think you misundertood my comments regarding prey biomass.  I did not request you to assess it as an index of fish trophic status. I understand that it is technically difficult as you replied.  What I requested was to discuss the possibility that the consideration of prey biomass may help to solve the gap between stomach content and stable isotope data, even though it is technically difficult, as with other hypotheses you provided.  You replied why it is technically difficult.  That would be by itself a perspective discussion, if it makes sense.

The question of the most efficient indices to apply to stomach content analyses for an accurate representation of the diet has been largely debated, is the subject of several meta-analyses and position papers (*e.g*. Hyslop 1980; Saika 2016; Amundsen and Sánchez‐Hernández, 2019; da Silveira et al., 2020). This question is still discussed, notably in the context of the implementation of the commercial species diet monitoring, to provide the data needed to move towards an ecosystemic management of fisheries. Nevertheless, even if discussions remain, most papers highlight that biomass-based indices are the most biased, and that considering abundance or occurrence is most cautious. We chose to follow this approach in our manuscript.

For example, biomass of remaining hard pieces (crustacean claws, fish otoliths etc.) cannot be easily converted into the actual biomass of the original prey consumed without accurate mass-mass or length-mass relationships. Including biomass as a descriptor of fish diet would also increase the well-known bias due do different prey digestibility, since the ratio between actual mass (i.e. the requested information) and measured mass of prey at different stages of degradation would not be similar. In other words, assessing the occurrence or abundance of preys is a testimony of what was actually consumed, notably as prey abundance is based on a parsimonious method that considers the lowest possible number of preys that originated the number of remains observed. But the weight of the items remaining in the stomach is the weight of the part not integrated in the predators’ tissues, while stable isotopes provide integrated information about the diet. Consideration of biomass may not necessarily resolve the observed gap between stomach contents and stable isotopes. For this reason, we have not included the consideration of biomass in perspective discussion.

We assume from editor’s repeated comment that this point was unclear in the manuscript. We thus reorganize the final part of the materials and methods section about stomach content to add a rationale for the use of abundance and occurrence rather than biomass.

L. 150-161 (on the manuscript without track changes): Abundance and occurrence-based indices were used as they are considered less biased than indices calculated from prey biomass measurement [12,45]. Assessing the occurrence or abundance of preys is a testimony of what was actually consumed, notably as prey abundance is based on a parsimonious method that limits overestimation of the number of degraded items, by estimating the most plausible number of prey individual that have originated the number of remains: paired items (*e.g.* otoliths, eyes, claws) were counted and divided by two. Other uncountable remains (*e.g.* muscles, gills, annelids bristles) were not counted and considered as remains of a single individual. On the contrary, biomass based indices are questioned, as they are more affect by well-known biases of stomach content. Differential digestibility of the preys leads to an overestimation of the mass of hard parts, and as biomass of parts found of the stomachs is the biomass of the undigested part of the diet, while stable isotopes reflect the part of the diet integrated in the tissues. Since stomach content analysis only provides a snapshot of fish diet, this approach was combined with stable isotopes analysis of fish tissue, which provides information on the time-integrated assimilated diet [35–37].

Other specific comments

**2.** L35: "trade-offs between foraging and mortality risk"
This can be misleading. The trade-off would occur between the relative benefits (foraging benefits relative to associated costs including mortality risk) of candidate resources, but not between foraging gains and associated cosits.

The sentence has been modified for a better understanding:

L. 36-37 (on the manuscript without track changes): “Consumers make trade-offs between the benefits (*e.g*., foraging) relative to associated costs including mortality”

**3.** L319: "smaller fish with low migration ability had higher δ15N values."
Are there any results or references to support this statement?

A reference was added to support the fact that juveniles had lower migrations abilities than adults (Cohen 1990, reference number 66 in the plain text). In addition, the sentence was slightly modified in the text:

L. 335-336 (on the manuscript without track changes): “because whiting migrate only after the first year of life [66], smaller fish with low migration ability had higher δ^15^N values.”


**4.** L339-340: In the EEC-SNS, winter nutrient concentration and temperatures were lower than in autumn.
Are there any results or references to support this statement?

EEC-SNS, temperature and nutrient concentration vary seasonally. Temperatures varied from 4°C in winter to 19°C in summer. In autumn, temperatures begin to decrease but remain higher than in winter. Similarly, in EEC-SNS, a phytoplankton bloom occurs in autumn. This bloom is less important than that of the spring period, but remains higher than in winter. Three references were added to support this statement (Breton et al. 2000, Carpentier et al. 2009, Morris et al. 2018, references 68, 69 and 70 in the plain text).

**5.** L354-355: Previous studies reported a particularly high energetic value for these species.
Please cite the papers.

Two references were added to support this statement (Spitz et al. 2010, Cresson et al. 2020, references 63 and 76 in the plain text)

**6.** Throughout Disucssion, I found several sentences not supported by any results or references.  Please provide supporting evidence for such assertive statements.

Some missing references to support our statements were added throughout discussion

- L. 297-298 (on the manuscript without track changes): “Most studies highlighted the consumption of crustaceans but did not distinguish between benthic or pelagic species [24,26,27]
- L. 317-318 (on the manuscript without track changes): “The positive relationship between δ^15^N values and body size is commonly observed for piscivores [13]»
- L. 335-336 (on the manuscript without track changes): “because whiting migrate only after the first year of life [66], smaller fish with low migration ability had higher δ^15^N values”
- L. 343 (on the manuscript without track changes): “Assuming high migration abilities for larger whiting [66]”
- L. 371-372 (on the manuscript without track changes): “Previous studies reported a particularly high energetic value for these species [63,76]”
- L. 396-397 (on the manuscript without track changes): One of the well-known limitations of stomach contents is the impossibility of identifying soft-bodied prey, such as fish eggs, due to their rapid digestion [44,79]

**Reviewers' comments:**

Reviewer's Responses to Questions

**Comments to the Author**

1. If the authors have adequately addressed your comments raised in a previous round of review and you feel that this manuscript is now acceptable for publication, you may indicate that here to bypass the “Comments to the Author” section, enter your conflict of interest statement in the “Confidential to Editor” section, and submit your "Accept" recommendation.

Reviewer #1: All comments have been addressed

Reviewer #2: All comments have been addressed

2. Is the manuscript technically sound, and do the data support the conclusions?

The manuscript must describe a technically sound piece of scientific research with data that supports the conclusions. Experiments must have been conducted rigorously, with appropriate controls, replication, and sample sizes. The conclusions must be drawn appropriately based on the data presented.

Reviewer #1: Yes

Reviewer #2: Yes

3. Has the statistical analysis been performed appropriately and rigorously?

Reviewer #1: Yes

Reviewer #2: Yes

4. Have the authors made all data underlying the findings in their manuscript fully available?

The [PLOS Data policy](http://www.plosone.org/static/policies.action#sharing) requires authors to make all data underlying the findings described in their manuscript fully available without restriction, with rare exception (please refer to the Data Availability Statement in the manuscript PDF file). The data should be provided as part of the manuscript or its supporting information, or deposited to a public repository. For example, in addition to summary statistics, the data points behind means, medians and variance measures should be available. If there are restrictions on publicly sharing data—e.g. participant privacy or use of data from a third party—those must be specified.

Reviewer #1: Yes

Reviewer #2: Yes

5. Is the manuscript presented in an intelligible fashion and written in standard English?

PLOS ONE does not copyedit accepted manuscripts, so the language in submitted articles must be clear, correct, and unambiguous. Any typographical or grammatical errors should be corrected at revision, so please note any specific errors here.

Reviewer #1: Yes

Reviewer #2: Yes

6. Review Comments to the Author

Please use the space provided to explain your answers to the questions above. You may also include additional comments for the author, including concerns about dual publication, research ethics, or publication ethics. (Please upload your review as an attachment if it exceeds 20,000 characters)

**Reviewer #1**: In this revision, the quality of this manuscript has greatly improved. Authors appropriately provided isotopic baseline in their study site and (fortunately) revealed that there were no significant variation in isoscape there. Overall, their revisions were satisfactory for me and I recommend to publish this paper once the other minor issues I listed below has been addressed.

**1.** Line 14-15: Please add a description of data collection for baseline organism.

The following sentence was added:

L. 15-16 (on the manuscript without track changes): “In addition, queen scallops (*Aequipecten opercularis*) samples were collected along with fish to be used as isotopic benthic baseline.”

**2.** Line 77: I suggest to replace “theoretically” with “empirically”.

Done

**3.** Line 78-79: This statement is not exactly true because some tissues like bone collagen enrich d13C greater than 1 %.

We recognize that for some tissues, the isotope discrimination may be lower or higher than 3.4 and 1‰. We have modified our sentence to focus on muscle, the tissue considered in our study.

L. 79-82 (on the manuscript without track changes): « The muscle nitrogen isotopic ratio (δ^15^N), empirically enriched by ~3.4 ‰ per trophic level, is classically used as an estimator of the trophic position [32]. Muscle carbon isotopic ratio (δ^13^C) is less enriched (~1 ‰ per trophic level), allowing its use as tracer of the origin of food sources [33].”

**4.** Line 120, “low lipid content”: This is ok but did you extract lipid from muscle sample? I couldn’t find the description about lipid extraction from the text. Lipid extraction is essential pretreatment for stable isotope analysis of muscle because adipose tissue generally has substantially decreased 13C values compared with muscle, and slight individual difference in the quantity of body fat can result in the difference of 13C.

Lipid content in fish muscle is generally low, but varies between fish species. The C:N ratio is used as an indicator of the lipid concentration in a sample. When lipid content is low, below 5% lipid (C:N<3.5), lipid correction – whether chemical extraction or mathematical correction of the isotopic ratios – is not requested (Post et al. 2007). In our dataset, C:N ratios ranged between 3.08 and 3.43 (average 3.19 ± 0.08), we did not performed lipid extraction pretreatment. The following sentence was added in the plain text:

L 122-126 (on the manuscript without track changes): White dorsal muscle is the most classical tissue used for stable isotopes analysis in fish because of its low turnover rate and low lipid content [43], notably for whiting, one of the species with lowest C:N ratios and consequently lipid content in the area [44]. As C:N ratios measured in all samples were low (average value: 3.19 ± 0.08; min: 3.08, max: 3.43), raw isotopic ratios were used.

**5.** Lines 152-153: Please include manufacture’s name and location for specific equipment.

Manufacture’s name and location were added for specific equipment

“Isotopic analyzes were carried out with a Thermo Fisher Scientific Delta V Plus mass spectrometer (Bremen, Germany), coupled to a Flash 2000 elemental analyzer with a Conflow IV interface (Thermo Scientific, Milan, Italy)” .

**6.** Line 162-164: This sentence should be moved to “Studied area and sampling”. Furthermore, detailed information about bivalve sample (e.g. sample size mean and SD of isotope ratios) should be included.

The following sentence was moved to the “Studied area and sampling” section.

L. 111-113 (on the manuscript without track changes): “Queen scallops (*A. opercularis*) samples were collected and stored frozen to be used as isotopic benthic baseline in the calculation of trophic level, following most classical approach [20,41,42]. “

Furthermore, additional information about bivalve sample was included in the material and method section of the manuscript:

L. 186-187 (on the manuscript without track changes): “A total of 76 isotopic measurements were used as a baseline for geostatistical interpolation (mean δ^15^N : 7.69 ± 0.90 ‰)”.

**7.** Line 186: I understood why you used 5 size class instead of quantitative body size data. Anyway, it may be helpful for readers to include some justifications about the use of size class in the text.

In the text, we include some justifications about the use of size class

L. 196-202 (on the manuscript without track changes): “Individual effects of these two explanatory variables, as well as their interactions were examined. Using discrete size classes in isotopic analyses GLMs was justified a posteriori by the non-monotonic character of the isotopic ratios versus size relationships. For stomach contents analyses, size-class analyses allow to account for the highly non-linear nature of dietary mechanisms. Each individual within a size class is considered as a replicate of the class. Having the diet of several individuals within a size-class is required to capture the diet of a class, and to limit the importance of extreme feeding mechanisms”.

**8.** Line 266, “… from -17.34 ± 0.68‰ (35+) to -16.77 ± 0.68‰ and -16.77 ± 0.65‰ (20-30cm) in winter.”: This sentence is confusing.

The sentence was modified for a better understanding:

L. 282-283 (on the manuscript without track changes): « from -17.34 ± 0.68‰ (35+) to -16.77 for both 20-25 (-16.77 ± 0.68‰) and 25-30 (-16.77 ± 0.65‰) size classes in winter.”

**9.** Lines 294-296: Again, did you extract lipids from whiting samples? If not, the interpretation of their 13C value is difficult because the difference of lipid concentration may explain the ontogenetic and seasonal difference of 13C values.

We have not extracted the lipids for the reason stated above (see answer number 5).

Moreover, as C:N ratios are similar for both seasons (3.18 ± 0.07 in autumn and 3.21 ± 0.09 in winter) and among size classes, influence of lipids on seasonal or ontogenetic differences can be considered negligible. Actual C:N ratios were missing and were added in the Table 1.

**10.** Fig. 4. Legends of 13C and 15N are reversed.

Corrected

**Reviewer #2**: The authors correctly addressed previous reviewers’ comments, and produced a sound manuscript that is acceptable for publication in PLOS one.
Below, I note a few errors:

**1.** Figure 3 is mislabeled: The left panel is 13C, whereas the central panel is 15N

Done

**2.** L. 198: change “analyzes” into analyses

Done

**3.** L267: change “increases” into increased

Done

7. PLOS authors have the option to publish the peer review history of their article ([what does this mean?](https://journals.plos.org/plosone/s/editorial-and-peer-review-process#loc-peer-review-history)). If published, this will include your full peer review and any attached files.


If you choose “no”, your identity will remain anonymous but your review may still be made public.


**Do you want your identity to be public for this peer review?** For information about this choice, including consent withdrawal, please see our [Privacy Policy](https://www.plos.org/privacy-policy).

Reviewer #1: No

Reviewer #2: No
